# Subjective stressors in school and their relation to neuroenhancement: a behavioral perspective on students’ everyday life “doping”

**DOI:** 10.1186/1747-597X-8-23

**Published:** 2013-06-18

**Authors:** Wanja Wolff, Ralf Brand

**Affiliations:** 1 Department of Sport and Exercise Psychology, University Potsdam, Am Neuen Palais 10, 14469, Potsdam, Germany

**Keywords:** Neuroenhancement, Stress, School, Doping

## Abstract

**Background:**

The use of psychoactive substances to neuroenhance cognitive performance is prevalent. Neuroenhancement (NE) in everyday life and doping in sport might rest on similar attitudinal representations, and both behaviors can be theoretically modeled by comparable means-to-end relations (substance-performance). A behavioral (not substance-based) definition of NE is proposed, with assumed functionality as its core component. It is empirically tested whether different NE variants (lifestyle drug, prescription drug, and illicit substance) can be regressed to school stressors.

**Findings:**

Participants were 519 students (25.8 ± 8.4 years old, 73.1% female). Logistic regressions indicate that a modified doping attitude scale can predict all three NE variants. Multiple NE substance abuse was frequent. Overwhelming demands in school were associated with lifestyle and prescription drug NE.

**Conclusions:**

Researchers should be sensitive for probable structural similarities between enhancement in everyday life and sport and systematically explore where findings from one domain can be adapted for the other. Policy makers should be aware that students might misperceive NE as an acceptable means of coping with stress in school, and help to form societal sensitivity for the topic of NE among our younger ones in general.

## Background

A researcher who chooses to pull an “all-nighter” to meet a submission deadline might be tempted to use Ritalin to prolong concentration and vigilance. This use of psychoactive substances to enhance one’s cognitive functioning, without the medical indication to do so, is prevalent [1-4]. For this behavior the term *neuroenhancement* (NE) has been coined [5]. NE is subject to ongoing debates in epidemiology and neuroethics. Issues of fairness and health have been emphasized in the light of a supposedly rising prevalence of NE among students [6]. It is important not to add to an untimely and unrealistic “media hype” at this stage of research however [7]. Most of the social science research published to date is aimed at describing NE prevalence and tends to neglect to ask *why* it might be prevalent. The representativeness of some epidemiological studies is at least questionable. Especially empirical studies on NE’s possible psychological roots are very scarce at the time.

This picture, including the extant problems with defining the phenomenon more consistently [8], is similar to what could be observed during the emerging years of social science research on doping in sport. Thus, one of the central claims of this article is that a projection of research strategies, fundamental assumptions, and underlying theories from the sporting domain may help to understand the psychology of NE.

Doping in sport is known to be associated with severe health consequences and in most competitive sports, it is seized with rigorous legal sanctions [9]. Both, health consequences and legal restrictions are also discussed with respect to NE [10,11]. Researchers have referred to a shared subjective “morality” of using performance enhancing substances in sport and everyday life [12]. With regard to drug abuse in everyday life, drug instrumentalization theory [13] postulates that non-addictive drug use can be explained by the individual’s expectation, that substances will facilitate performance in previously learned behaviors [14,15]. Similar arguments have been introduced to the doping literature [16]. With regard to both domains, taking goal system theory [17] as an exemplary theoretical framework, the use of a performance enhancing substance represents a functional means to achieve a highly valued end. In the sporting domain, erythropoietin (EPO) is employed for improving one’s athletic endurance, making winning more probable. In the NE domain, methylphenidate (e.g., in Ritalin) can be used as a means to prolong concentration during learning sessions [13], thus facilitating the end of academic success. It is a worthwhile hypothesis that basic psychological representations of these two behaviors are therefore similar.

Attitudes are defined as the individual’s subjective evaluation of a person or object, and represent such a basic psychological representation [18]. Attitudes have been shown to be amongst the strongest predictors for doping [19], and doping specific attitude scales have been shown to predict doping intentions and behavior [20]. Attitudes are a promising target construct for NE researchers not only because they can predict behavior; but also because they might form before this behavior occurs. It is true that attitudes may also result from previous behavioral choices [21]. However, to prevent a behavior from occurring, changing peoples’ attitudes has been shown to be one of the superordinate goals of preventative interventions in the doping domain [22]. Our proposition is to measure NE attitudes with an adapted version (domain specific enhancement scale) of an internationally validated doping attitude scale therefore.

Another parallel between NE and doping in sport might be seen in the use of performance enhancing substances in order to cope with stress and anxiety. With regard to doping in sport, authors have proposed that athletes are more likely to use illicit substances if demands are high and they appraise their own resources as insufficient to cope with these demands [16]. Alcohol is one substance, which is employed by students to reduce experienced pressure to perform [23]. More recent exploratory studies have also found correlations between global psychological distress in college students and NE [24]. One study even concludes that “some students take drugs to manage the current vast study demands ([2]; p. 268)”. This study did not include measures to explicitly assess these demands however. It is a worthwhile hypothesis that diverse NE substances may be employed as coping means.

With respect to which substances might be regarded as being suitable as coping means, we argue for a *behavioral definition of NE*. From this viewpoint, NE aims at the enhancement or rebuilding of cognitive performance (means-to-end relation), thus representing one of the nine instrumentalization goals [13,15] attributed to systematic non-addictive drug use (the others are: improved social interaction, facilitated sexual behavior, facilitated recovery from and coping with psychological stress, euphoria and hedonia, improved physical appearance and attractiveness, self-medication for mental problems, sensory curiosity and facilitating spiritual and religious activities). This behavioral viewpoint *neither* implies that the substance is a prescription drug or not (for problems with the substance-based definition of doping in sport see [25]), *nor* that it is effective (for the often overestimated expectations regarding typical NE substances see [26]), *nor* that it could not be used for resolving other annoying states (e.g., indulgence or boredom). Our behavioral viewpoint thus includes the first claim made by drug instrumentalization theory (substances are used as instruments) but does not necessarily imply the second one (substances increase performance through changes in mental states). The *assumed functionality* of the behavior is its key definitional aspect: If a person consumes a substance with the goal to improve cognitive performance, and if he or she assumes (subjectively expects) that this substance is able to improve his or her cognitive performance, this person is neuroenhancing. For example, caffeinated drinks (“energy drinks”) are consumed frequently among younger people [27,28]. If such drinks are explicitly consumed to enhance cognitive performance they are means to an end; and therefore qualify as neuroenhancers. Epidemiological evidence on the use of such lifestyle neuroenhancers is widely lacking (for one exception see [29]).

While we focus on the assumed functionality of NE, it has to be noted that the actual effects of enhancing substances vary greatly between individuals [30]. Enhancers have shown to be more effective in novel situations [31] and for participants with poor memory capacity [32] for example.

With regard to substances we propose three NE variants: *lifestyle drug NE* (e.g., high dosed caffeinated drinks), *prescription drug NE* (e.g., Methylphenidate) and *illicit substance NE* (e.g., Cocaine). For lifestyle drug NE, substances like coffee or caffeinated drinks, lifetime prevalence in students has been reported to be 53.2% and 39% respectively [29]. Lifetime prevalence of prescription drug and illicit substance NE among US American students (University and College) have been found to range between 7 and 9% [6,33,34]. One recent study, which has employed a randomized response technique in order to better account for social sensitivity of the issue, suggests the one-year prevalence for prescription and illicit drug NE among German students to be as high as 20% [4].

The advantage of distinguishing between these three NE variants is (at least) twofold. Firstly it allows for a much more faceted view of the phenomenon, and its behavioral basis respectively. Secondly, it allows for describing multiple NE substance abuse. This is relevant, as lifestyle drug NE may pave the road for later prescription drug or illicit substance NE (similar to the behavioral gateway hypothesis in the doping in sport literature, [35]). A few studies in the doping domain have already investigated the (mental) relation of doping and recreational drugs [36-38] or nutritional supplements [38]. The extant literature on NE has not drawn on this possible link as yet.

Based on these assumptions our empirical hypotheses are as follows:

Hypothesis 1 (*multiple NE substance abuse*): Use of lifestyle drug NE is associated with a higher probability of prescription drug and illicit substance NE.

Hypothesis 2 (*domain specific enhancement attitude*): Self-reported use of lifestyle, prescription drug and illicit substance NE can be predicted by a domain specific (i.e., NE) variant of a doping attitude scale*.*

Hypothesis 3 (*NE and stress in school*): Pressure to perform, test anxiety and overwhelming demands can be used to incrementally (over and above the effects of age and gender; see below) predict the three NE variants.

## Methods

### Setting, sample and procedure

The study was conducted in a vocational school in Germany (students must be graduated from secondary school, therefore having at least ten years of prior education, if they choose to enroll in a vocational school) Participants were 519 students (73.1% female), representing a self-selected sample (61.1%) from this school’s 850 students. Mean sample age was 25.8 ± 8.4 years. Participants filled out a paper and pencil questionnaire anonymously during class. All participants gave full informed consent prior to the measurement. Ethical approval for the study was granted by the University of Potsdam.

### Measures

Participants were asked whether or not they have ever used an NE substance (“Have you ever used a substance with the goal to increase your cognitive performance”) on separate questionnaire pages for lifestyle, prescription drug and illicit substance NE. Emphasis was laid on the functional aspect of consumption so that participants would not report for example their morning cup of coffee. The most prevalent substances for lifestyle NE (e.g., functional use of coffee, caffeine pills, creatine, energy drinks) and prescription drug NE (e.g., methylphenidate, modafinil, beta-blocker) were listed as examples. Because of social sensitivity issues, no list was provided for illicit substance NE. Above that, participants had to indicate their frequencies of use, separately for lifestyle and prescription NE (not for illicit substance NE) on a 6-point Likert-type scale from 0 = “never” to 5 = “several times a day”.

*Pressure to perform* was assessed using the revised [39] 5-item version of the Pressure-to-Perform-Scale [40]. *Test anxiety* was assessed using the [39] 5-item version of the Test Anxiety Inventory [41]. Both instruments showed sufficient psychometric properties in previous studies. In our sample these scales reached internal consistencies (Cronbach’s -α) of .76 and .87 respectively. *Overwhelming demands in school* was assessed with the single question *“*Are you overwhelmed by the demands in school”. Answers had to be given on a 5-point Likert-type scale (ranging from 1, “I don’t feel overwhelmed” to 5, “I do feel overwhelmed”). *NE attitude* was assessed using a modified 9-item version (items 1, 2, 4, 6, 7, 11, 13, 15 and 16) of the Performance Enhancement Attitude Scale [20]. These items were adapted by replacing doping related terminology with that of neuroenhancement, and the context of sport performance to that of academic performance. This adapted scale will be labeled NEAS throughout this manuscript. Its internal consistency was α = .79 in our sample. Factorial structure of the NEAS is provided as supplemental material [Additional file 1].

### Statistical analyses

The hypothesis on multiple NE substance abuse (H1) was tested with a χ^2^-test. To test the domain specific NE attitude (H2) and the NE and stress in school (H3) hypotheses, six stepwise multiple logistic regression analyses were conducted, with either lifestyle, prescription drug or illicit substance NE as dependent variables. For testing H2 and H3 we conducted two stepwise multiple linear regression analyses with lifestyle NE frequency being the dependent variable. Step one is to control for the variables sex and age (as these two variables are known to make a difference in NE behavior; [4]). In step two either the *NE attitude* (to test the domain specific enhancement attitude hypothesis; H2), or *test anxiety*, *pressure to perform* and *excessive demands* is included (*NE and stress in school hypothesis; H3*).

## Results

Descriptive statistics as well as detailed results of all linear regression analyses are depicted in Tables 1 and 2.

**Table 1:** Descriptive statistics

	***M***		***SD***		
NEAS	2.80		0.86		
Pressure to Perform	2.91		0.77		
Test Anxiety	2.65		1.01		
Overwhelming Demands	2.36		0.96		
	Prevalence				
	Yes			No	
Prescription-drug NE	8.00%			92.00%	
Illicit-substance NE	8.80%			91.20%	
Lifestyle-drug NE	62.60%			37.40%	
Lifestyle-drug NE:	monthly	weekly	twice a week	daily	several times a day
Frequency of use^a^					
	14.90%	10.90%	9.70%	19.20%	45.30%

^a^ Percentage only from those who have admitted lifestyle-drug NE.

**Table 2:** Results of linear regression analyses predicting frequency of lifestyle NE use

**Enhancement attitude (H2)**				**Stress in school (H3)**			
**Predictor**^**a**^	**Δ*R***^***2***^	**β**	***df***	**Predictor**^**a**^	**Δ*R***^***2***^	**β**	***df***
Step 1	.006		2, 428	Step 1	.005		2, 432
Sex		-.07	427	Sex		-.06	431
Age		.05	427	Age		.04	431
Step 2	.027**		1, 427	Step 2	.034**		3, 429
NEAS		.17**	426	Pressure to Perform		-.11	428
				Test Anxiety		.02	428
				Overwhelming Demands		.22**	428
Total *R*^*2*^	.034**		3, 427		.039**		5, 429
Total *F*	18.03**				12.48**		
*n*^*b*^	430				434		

^a^ Test statistic for significance testing of all *R*^2^’s and Δ*R*^2^’s is the *F*-statistic, test statistic for significance testing of all β’s is the *t*-statistic.

^b^ Differences in *n* and test scores for step one are due to missing values in the respective analyses. ** *p* < .01.

### Multiple NE substance abuse

For prescription drug and illicit substance NE both χ^2^-tests were significant, χ^2^(1) = 12.08, *p* < .01, and χ^2^(1) = 17.96, *p* < .01 respectively. Odds ratios illustrate that users of lifestyle drug NE had a higher chance of prescription drug (*OR* = 4.72, *CI95* = 1.82 - 12.24) and illicit substance use, *OR* = 8.75, *CI95* = 2.66 - 28.73. These results support the multiple NE substance abuse hypothesis.

### Domain specific enhancement attitude

The omnibus tests of model coefficients for step-one of the logistic regression models were significant for lifestyle drug (χ^2^(2) = 14.74, *p* < .01) and illicit substance NE, χ^2^(2) = 18.35, *p* < .01. The test for prescription drug NE was not significant, χ^2^(2) = 1.69, *p* = .43. Age significantly predicted lifestyle drug NE (*OR* = 0.96, *p* < .01, *CI95* = 0.93 - 0.98) and males were more likely to use illicit substances, *OR* = 3.98, *p* < .01, *CI95* = 2.01 - 7.77. No significant results were found for the remaining associations of age and gender with the other NE variants (all *p’s* ≥ .11) and the frequency of lifestyle drug NE, all *p*’s ≥ .16. Because of a lack in statistical power (due to the few “yes”-responses obtained) frequency of use data for prescription drug NE could not be analyzed with linear regression analysis.

The omnibus tests of model coefficients for step-two of the logistic regression models were significant for lifestyle drug (χ^2^(1) = 35.68, *p* < .01), prescription drug (χ^2^(1) = 22.71, *p* < .01) and illicit substance NE, χ^2^(1) = 28.89, *p* < .01. NEAS explained incremental variance (over and above the effects of age and gender) in the probability of lifestyle drug (*OR* = 2.11, *p* < .01, *CI95* = 1.62 - 2.74), prescription drug (*OR* = 2.67, *p* < .01, *CI95* = 1.76 - 4.03) and illicit substance NE, *OR* = 2.98, *p* < .01, *CI95* = 1.96 - 4.55. Above that, NEAS scores were significant predictors for the frequency of lifestyle drug NE. This data support the hypothesis, that frequency of NE can be predicted by NE attitude, as measured by the domain specific (NE) enhancement attitude scale NEAS.

### NE and stress in school

Regression models for lifestyle drug, and illicit substance NE remained both insignificant, χ^2^(3) = 4.09, *p* = .25, and χ^2^(3) = 2.78, *p* = .43. Yet the omnibus test for prescription drug NE was significant, χ^2^(3) = 14.98, *p* < .01. *Overwhelming demands* was the only predictor to explain incremental variance (over and above the effects of age and gender) in this model*, OR* = 2.30, *p* < .01, *CI95* = 1.39 - 3.79. The same variable, *overwhelming demands* was the only significant predictor for frequency of lifestyle drug NE (Table 2). This data provides partial support for our hypothesis on NE and stress in school.

## Discussion

This study found support for the hypotheses that lifestyle drug NE is associated with prescription drug, as well as illicit substance NE (multiple substance abuse). Further, the hypothesis that NE can be predicted by an adapted doping attitude test (NEAS) was supported. Prescription drug and the frequency of lifestyle substance NE could be predicted by overwhelming demands in school.

We did not aim at measuring prevalence rates (this would not have been reasonable with a self-selected sample). Yet it speaks in favor of the validity and generalizability of the reported results, that in our sample the observed frequencies of use of prescription drugs and illicit substances were similar to the ones reported in previously published studies [6,29,33,35].

Compared to non-users, lifestyle drug NE users were more likely to be prescription drug and illicit substance users. This pattern of multiple NE abuse mirrors the phenomenon of polydrug use in substance addiction. Polydrug use is prevalent and has a strong impact on public health [42]. Research findings as well as preventative or rehabilitative strategies of dealing with this phenomenon can possibly be adapted to the NE domain.

Neither pressure to perform nor test-anxiety was correlated with any of the three NE variants. However, overwhelming demands explained incremental variance in prescription, as well as in frequency of lifestyle drug NE. This finding supports the stress theoretical claim that a situation has to be appraised as overwhelming to cause stress and maladaptive coping strategies. Preventative accounts should therefore focus on individuals, who explicitly state to be in a situation that by far exceeds their coping resources.

Further, the other stressors’ lack of incremental validity leads us to propose the hypothesis that NE might at least partly reflect some kind of a lifestyle aspect. This would call for an even broader construct of enhancement. It is impossible to rule out that further conceptual issues (e.g., specific drugs asked for; method artifacts) could have accounted for the absence of these results. We think that future studies should investigate also the possibility of stress-independent enhancement and psychological variables associated with it.

We have argued that NE and doping in sports draw back on similar psychological representations about enhancement behavior. As expected, the adapted (NE domain specific) doping attitude scale (NEAS) was able to predict NE. Exponential beta coefficients (odds ratios) of NEAS were lowest for lifestyle drug and highest for illicit substance NE. This ordinal rank is comparable to the legal supplement to illegal doping rank order in sport. Building on this interpretation we suggest that *enhancement* (everyday life as well as doping in sport) can be conceptualized along the two orthogonal dimensions *health* and *sanctions*. This conceptualization could predict closer proximity of illicit NE and doping (severe sanctions and severe health consequences) compared to prescription drug NE (sanctions and health consequences moderate to severe) and lifestyle drug NE (no sanctions and low to moderate health consequences), and thus explain higher incremental validity of NEAS scores in this domain. This orthogonal conceptualization has the advantage of taking the dimensions’ relative independence into account (e.g., legality depends on jurisdiction, and health consequences depend on the individual’s substance specific lay knowledge). Future studies should test this hypothesis, and maybe include additional dimensions such as lifestyle (see above) or functionality to draw an even closer description of the behavior.

### Limitations

Although all students of the selected vocational school were asked to fill out the questionnaire, 32% did not participate. Not knowing who chose not to participate, selective attrition cannot be ruled out. Hence we again emphasize that our study does not represent an epidemiological account on NE prevalence. Still, given the above reported, comparable NE prevalence rates from other studies, we propose that our sample sufficed for investigating the proposed research question: The relationship of psychological constructs and NE behavior.

Unfortunately, we did not assess the use and patterns of one distinct and widely used drug, which is often consumed to better cope with psychological demands: Alcohol [43]. It is possible that students, who use alcohol to cope with stress, might be less likely to utilize other substances, for example illicit drugs. We further did not assess addiction-like use patterns. Addiction-like use could deflate the predictor-NE associations we have found. This possible moderating and deflating effects should be addressed in future studies.

### Implications

The present study advances the field of NE research by focusing on NE behavior. With regard to their psychological bases, neuroenhancement in everyday life and doping in sport may be comparable phenomena. Even after our study, in which we have drawn conceptual parallels, researchers should directly (empirically) investigate into these similarities. To arrive at causal explanations then, for example on possible gateways from NE in everyday life to doping in sport (or the other way round), prospective studies and experimental designs are urgently needed.

Policy makers might want to address school environment factors since overwhelming demands seem to be related to NE behavior. Even more, they should be aware that students might misperceive NE as an acceptable means of coping with stress in school, and help to form societal sensitivity for the topic of NE among our younger ones in general.

## Competing interests

The authors declare that they have no competing interests.

## Authors’ contributions

WW and RB designed the study. WW conducted the statistical calculations and wrote the first draft of the manuscript. RB revised the first draft and conducted the statistical analyses for the supplemental material. Both authors then jointly worked on all subsequent versions of the manuscript. Both authors read and approved the final manuscript.

## Supplementary Material

Additional file 1 Factorial structure of the Neuroenhancement Attitude Scale (NEAS). Click here for file
