# Supplementary material for: Subjective stressors in school and their relation to neuroenhancement: a behavioral perspective on students’ everyday life “doping”
Source: Subst Abuse Treat Prev Policy. 2013 Jun 18;8:23. doi: 10.1186/1747-597X-8-23 (PMC3698138; doi:10.1186/1747-597X-8-23)
Supplement: Additional file 1 — Factorial structure of the Neuroenhancement Attitude Scale (NEAS). [file 1747-597X-8-23-S1.docx]

Supplementary material for the manuscript entitled:

Subjective stressors in school and their relation to neuroenhancement: A behavioral perspective on student’s “everyday life” doping

Factorial structure of the *Neuroenhancement Attitude Scale* *(NEAS)*

Wanja Wolff and Ralf Brand

University of Potsdam

The *Neuroenhancement Attitude Scale* *(NEAS)*, is an adaptation of the *Performance Enhancement Attitude Scale (PEAS)*, as proposed by Andrea Petroczi and Eugene V. Aidman [1]. The PEAS represents an international standard for measuring doping attitudes of athletes in sport [2-6]. Imitating the factorial structure of the PEAS, the NEAS is also supposed to be unidimensional with regard to its factorial structure. The following material is to illustrate some basic psychometric characteristics of the NEAS scale.

##### Basic psychometric characteristics

**Item construction**

Nine PEAS items with high face validity for the behavioral domain of neuroenhancement were preselected for the NEAS, and the wording was switched from the doping in sport to the neuroenhancement domain.

**Sample description**

The study was conducted in a vocational school in Germany (students have to be graduated from secondary school, therefore having at least ten years of prior education, if they choose to enroll in a vocational school, in order for specific vocational training). Participants were 519 students (73.1% female), representing a self-selected sample (61.1%) from this school’s 850 students. Mean sample age was 25.8 ± 8.4 years. Participants filled out a paper and pencil questionnaire anonymously during class. All participants gave full informed consent prior to the measurement.

**Internal consistency and structure of the scale**

The NEAS’s internal consistency was α = .79. None of the corrected item-total correlations was lower than *r_it_* = .32. Together with a moderately high item inter-correlation coefficient (*r_ii_* = .31) and a moderately low value for item-precision (*P_α_* = .02), these results point to a homogenous set of items and indicate an adequate measurement accuracy of the scale (see table 1 for complete item statistics).

Though unintended, it is still possible that subsets of items represent different components (e.g., *legality* vs. *health*) that should be addressed by calculating separate sub-scores. A significant Bartlett-test, χ^2^(*df* = 36) = 992.18 and the adequate KMO-coefficient of .85 allow to explore this possibility by principal component analysis. The criterion for factor extraction was set at λ > 1. As a result there is an explained amount of variance of 39.94% loading on only one (λ = 3.60) extracted component (table 1 for item communalities and item-factor loadings). Velicer’s Minimum Average Partial test [7] recommends the extraction of only one factor (with at proposed factors 1 and 2 being .03 and .05) as well.

These results indicate that the NEAS scale can be regarded to measure a unidimensional construct.

**Score distribution**

The sample’s mean NEAS score was *M* = 2.80, with a standard deviation of *SD* = 0.86 (Figure 1). Excess (*z* = 0.28, *SE* = 0.11) and kurtosis (*z* = 0.08, *SE* = 0.22) indicate an adequate score distribution.

**NEAS validity**

We compared the users’ with the non-users’ mean NEAS scores separately for lifestyle-drug, prescription drug and illicit-substance NE [8]. The NEAS scores of subjects who reported to have used any of the three NE variants were significantly higher than the non-users’ scores. The results of this statistical analysis are given in table 2. Albeit weak, this is a preliminary indicator for the NEAS’s criterion validity.

**References**

1. Petróczi A, Aidman EV: **Measuring explicit attitude toward doping: review of the psychometric properties of the Performance Enhancement Attitude Scale.** *Psychology of Sport and Exercise* 2009, **10**(3):390-396.
2. Backhouse, SH, Whitaker L, Petróczi A: **Gateway to doping? Supplement use in the context of preferred competitive situations, doping attitude, beliefs, and norms.** *Scandinavian Journal of Medicine & Science in Sports* 2011, e published ahead of print.
3. Zelli A, Mallia L, Lucidi F: **The contribution of interpersonal appraisals to a social-cognitive analysis of adolescents' doping use.** *Psychology of Sport and Exercise* 2010, **11**(4):304-311.
4. Uvacsek M, Nepusz T, Naughton DP, Mazanov J, Ránky MZ, Petróczi A: **Self‐admitted behavior and perceived use of performance‐enhancing vs psychoactive drugs among competitive athletes.** *Scandinavian Journal of Medicine & Science in Sports* 2011, **21**(2):224-234.
5. Moran A, Guerin S, Kirby MK, MacIntyre T: **The development and validation of a doping attitudes and behaviour scale.** *Report to WADA & Irish sports council* 2008.
6. Petróczi A, Uvacsek M, Nepusz T, Deshmukh, Shah I Aidman EV, Barker J, Tòth M, Naughton D: **Incongruence in doping related attitudes, beliefs and opinions in the context of discordant behavioural data: in which measure do we trust?** *PLoS One* 2011, **6**(4):e18804.
7. **O’Connor BP: SPSS and SAS programs for determining the number of components using parallel analysis and Velicer’s MAP test.** *Behavior Research Methods, Instruments & Computers* 2000, **32**(3):396-402.
8. Wolff W, Brand R: **Subjective stressors in school and their relation to neuroenhancement: a behavioral perspective on students’ everyday life “doping”.** *Substance Abuse Treatment, Prevention, and Policy* 2013, **8**:23

Table 1

*Supplementary material:* *Items, descriptive item statistics, and results of principle component analysis (exploratory) of the NEAS.*

|  | Item descriptives | | *Principal component analysis* | | |
| --- | --- | --- | --- | --- | --- |
|  | *M* (*SD*) | Excess Kurtosis | *r_it_* | *h^2^* | *a_C1_* |
| 1. Neuroenhancement is necessary to be competitive. | 2.25 (1.23) | 0.80 0.06 | .54 | .46 | .68 |
| 1. Neuroenhancement is no cheating. | 3.20 (1.55) | 0.17 -0.94 | .47 | .34 | .59 |
| 1. Only the quality of performance should matter, not the way one achieves it. | 3.17 (1.57) | 0.29 -0.93 | .45 | .31 | .56 |
| 1. In today’s society one is pressured to take performance-enhancing drugs. | 2.60 (1.44) | 0.65 -0.67 | .57 | .50 | .71 |
| 1. Students who take recreational drugs, use them because they help them in competitive situations. | 3.11 (1.34) | 0.08 -0.76 | .42 | .30 | .55 |
| 1. Cognitive enhancing drugs give the motivation to learn, work and perform at the highest level. | 3.06 (1.29) | 0.20 -0.53 | .65 | .59 | .77 |
| 1. Neuroenhancement is an unavoidable part of learning and working. | 2.13 (1.23) | 0.97 0.23 | .59 | .53 | .73 |
| 1. Health problems related to stress and overload are just as bad as those from neuroenhancement. | 3.53 (1.53) | -0.09 -1.01 | .32 | .18 | .42 |
| 1. Legalizing neuroenhancers would be beneficial. | 2.19 (1.29) | 1.05 0.56 | .49 | .39 | .63 |

*Note.* Range is 1 = *strongly disagree* to 6 = *strongly agree* for all items


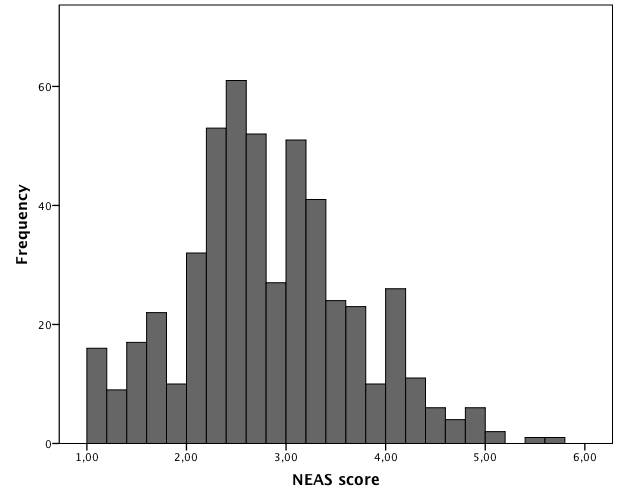


Figure 1 Supplementary material: Distribution of NEAS scores

| Table 2  *Differences in NEAS mean scores for users compared to non-users* | | | | | | | | |
| --- | --- | --- | --- | --- | --- | --- | --- | --- |
|  | Lifestyle-drug NE | |  | Prescription-drug NE | |  | illicit-substance NE | |
|  | *M* | *SD* |  | *M* | *SD* |  | *M* | *SD* |
| User | 2.99 | 0.85 |  | 3.48 | 0.83 |  | 3.49 | 0.91 |
| Non-user | 2.47 | 0.76 |  | 2.74 | 0.85 |  | 2.72 | 0.82 |
| *t* | 6.96*** | |  | 5.32*** | |  | 5.88 | |
| *df* | 500^a^ | |  | 500 | |  | 486 | |
| *Cohens d* | 0.65 | |  | 0.88 | |  | 0.89 | |
| *Note.* ^a^ Differences in *df* are due to missing values in the respective analyses.  *** *p* < .001 | | | | | | | | |
